# Disease activity flares and pain flares in an early rheumatoid arthritis inception cohort; characteristics, antecedents and sequelae

**DOI:** 10.1186/s41927-019-0100-9

**Published:** 2019-11-18

**Authors:** Daniel F. McWilliams, Shimin Rahman, Richard J. E. James, Eamonn Ferguson, Patrick D. W. Kiely, Adam Young, David A. Walsh

**Affiliations:** 1 Pain Centre Versus Arthritis, University of Nottingham, Clinical Sciences Building, City Hospital, Nottingham, NG5 1PB UK; 2 Division of ROD, University of Nottingham, Clinical Sciences Building, City Hospital, Nottingham, NG5 1PB UK; 3 NIHR Biomedical Research Centre, University of Nottingham, Clinical Sciences Building, City Hospital, Nottingham, NG5 1PB UK; 4 School of Psychology, University of Nottingham, Nottingham, UK; 5 Department of Rheumatology, St George’s University Hospitals NHS Foundation Trust, London, UK; 6 University of Hertfordshire, Hatfield, UK; 7 Department of Rheumatology, Sherwood Forest Hospitals NHS Foundation Trust, Sutton-in-Ashfield, UK

**Keywords:** Early rheumatoid arthritis, Flares, Inflammation, Pain, Disability

## Abstract

**Background:**

RA flares are common and disabling. They are described in terms of worsening inflammation but pain and inflammation are often discordant. To inform treatment decisions, we investigated whether inflammatory and pain flares are discrete entities.

**Methods:**

People from the Early RA Network (ERAN) cohort were assessed annually up to 11 years after presentation (*n* = 719, 3703 person-years of follow up). Flare events were defined in 2 different ways that were analysed in parallel; DAS28 or Pain Flares. DAS28 Flares satisfied OMERACT flare criteria of increases in DAS28 since the previous assessment (≥1.2 points if active RA or ≥ 0.6 points if inactive RA). A ≥ 4.8-point worsening of SF36-Bodily Pain score defined Pain Flares. The first documented episode of each of DAS28 and Pain Flare in each person was analysed. Subgroups within DAS28 and Pain Flares were determined using Latent Class Analysis. Clinical course was compared between flare subgroups.

**Results:**

DAS28 (45%) and Pain Flares (52%) were each common but usually discordant, with 60% of participants in DAS28 Flare not concurrently in Pain Flare, and 64% of those in Pain Flare not concurrently in DAS28 Flare. Three discrete DAS28 Flare subgroups were identified. One was characterised by increases in tender/swollen joint counts (14.4%), a second by increases in symptoms (13.1%), and a third displayed lower flare severity (72.5%). Two discrete Pain Flare subgroups were identified. One occurred following low disease activity and symptoms (88.6%), and the other occurred on the background of ongoing active disease and pain (11.4%). Despite the observed differences between DAS28 and Pain Flares, each was associated with increased disability which persisted beyond the flare episode.

**Conclusion:**

Flares are both common and heterogeneous in people with RA. Furthermore our findings indicate that for some patients there is a discordance between inflammation and pain in flare events. This discrete flare subgroups might reflect different underlying inflammation and pain mechanisms. Treatments addressing different mechanisms might be required to reduce persistent disability after DAS28 and Pain Flares.

## Background

Flare events are a common experience for people with chronic conditions, including those with rheumatoid arthritis (RA). RA flares are often described by reference to joint swelling, pain and fatigue, and interpreted as deteriorations in inflammation [1]. RA flare has been defined as ‘a cluster of symptoms of sufficient duration and intensity that cannot be self-managed by the patient and require initiation, change or increase in therapy’ [2]. The OMERACT initiative classified RA flare based upon increases in the 28 joint disease activity score, DAS28 [3]. DAS28 is widely used in clinics, has validity for treatment targets and shaping long-term outcomes [4]. These DAS28-based flare criteria exceed 70% specificity and sensitivity, compared with the judgement of the patient [3]. They also showed sensitivity of 88–100%, and specificity of 57–65% for detecting investigator-defined flares and after biologic discontinuation in 1 clinical trial [5]. There is evidence that flares contribute to worsening cardiovascular morbidity, joint damage and other long-term outcomes [6]. Fear of flares might induce wide-ranging behavioural modifications, including retreat from social life and reductions in physical activities over long periods of time [7]. RA flares are commonly treated with anti-inflammatory glucocorticosteroids and changes to disease modifying anti-rheumatic drugs (DMARDs).

Increased pain is a common feature of RA flares. Pain in RA might be driven by inflammatory disease activity, but might also result from other mechanisms including central sensitisation. Diverse pain mechanisms lead to discordance between symptom report, physician report and inflammatory parameters [8, 9]. Non-inflammatory musculoskeletal pain can also be subject to periodic increases in severity, for example in osteoarthritis [10] or fibromyalgia [11]. Not all flares are associated with noticeable joint swelling [7, 12], and flare severity in RA might not necessarily reflect the degree of increase in inflammatory disease activity. Misinterpretation of RA flares as indicative of uncontrolled inflammatory disease could lead patients to be exposed to risks from interventions which are either unnecessary or ineffective, and might be a barrier to more effective management options.

We hypothesised that amongst people who are experiencing RA flares, discrete groups might be identified with increased inflammatory disease activity or activation of non-inflammatory pain mechanisms. We explored two types of flare that might require distinct, personalised, treatment; DAS28 Flares and Pain Flares. We investigated, using Latent Class Analysis (LCA) [13], possible heterogeneity within DAS28 and Pain Flares, and report sequelae of discrete flare subgroups in terms of the key RA outcomes of DAS28, pain and disability.

## Methods

### Patients

Data were from the Early RA Network (ERAN), an inception cohort recruited from 2002 to 2013 and followed up until study end in 2013 [14]. Data in the ERAN study were collected from 22 outpatient rheumatology centres in UK and Ireland. All participants gave signed, informed consent to participate in line with the Declaration of Helsinki. The ERAN study was approved by Trent Research Ethics Committee (reference 01/4/047). Recruitment was at the first diagnosis of RA by a rheumatologist, and follow up visits were at 3–6 months and then annually after baseline. The eligible population for this study were people who had 2 consecutive DAS28-ESR scores and 2 consecutive SF36-Bodily Pain measures reported during baseline and follow up. The consecutive DAS28-ESR and SF36-Bodily Pain values could be taken from any eligible time points. To avoid multiple counting, only one DAS28 and one Pain Flare episode was investigated per patient (the first documented flare of each type after recruitment to the cohort). The eligible population for this study was 719 people, covering 3703 person-years (median follow up 5 years, IQR 3 to 7).

### Data collection

Participants attended study visits in outpatient departments at each centre. Data were collected for age, gender, body mass index (BMI), smoking, symptom duration (months), serology, DAS28 (erythrocyte sedimentation rate (ESR), swollen joint count (SJC), tender joint count (TJC), visual analogue scale (VAS) for global disease, HAQ disability, SF36 quality of life (norm-transformed for Bodily Pain (BP), Vitality (VT) and Mental Health (MH) subscales) [15].

### Flare classification

DAS28 Flare and Pain Flare were derived separately. To avoid multiple counting, the first DAS28 Flare and the first Pain Flare after baseline were each selected for analyses. DAS28 Flares were classified if there was an increase between consecutive study visits in DAS28 ≥ 1.2; or an increase ≥0.6 if the first of the paired visits had DAS28 ≤ 3.2 [1, 2]. Pain Flares were classified if there was a worsening ≥4.8 points of non-normed SF36-BP between consecutive measurements. This was derived using the mean increase in SF36 Bodily Pain score associated with a 1.2 point increment in DAS28 at baseline. The linear regression coefficient between DAS28-ESR vs SF36-Bodily Pain at baseline was − 3.96 (se, 0.23).

Flare severities were measured as change in each variable from the previous time point to the flare event. The term “before” refers to the single measurement at the assessment immediately preceding the designated flare; and “after” refers to the single measurement immediately following. If no data were available at the before or after time points, then the data were classified as missing.

### Statistical analysis

Potential heterogeneity within DAS28 or Pain flares experience were investigated by Latent Class Analysis (LCA) of change scores for ESR, SJC, TJC, VAS, SF36-BP, SF36-VT, SF36-MH to search for flare subgroups. Several characteristics of the LCA models were assessed to provide guidance for the number of latent classes that were selected [16]. An initial estimate was made for the amount of information lost by the simplest LCA model. Diagnostic indices Akaike Information Criteria (AIC), Bayesian information Criteria (BIC), sample size adjusted BIC (ssBIC) were derived for a 1 class model (ie. no subgroups). Then incremental, iterative increases in the number of classes were employed [17, 18]. A comparison was made between increasing numbers of classes for each of the diagnostic indices (AIC, BIC and ssBIC), plus entropy values, and the improvement compared to the previous model (assessed by *p* values from Vuo-Lo-Mendell-Rubin likelihood ratio test (VLMR) and Bootstrap likelihood ratio test (BLRT)) [8, 16]. A lower limit of 5% subgroup size was set for LCA, to give meaningful classes. Each index, test or measurement gave information regarding the preferred model which best explained and fitted the flare severity data. Smaller values of AIC, BIC and ssBIC as the number of classes increased was considered desirable, indicating less information loss. Entropy (range 0–1) reflected the overall probability that cases were assigned to the correct latent class, and higher values indicate less uncertainty. LMR-LRT and BLRT were tests for goodness of fit compared to the previous model, and low *p*-values indicated a likely improvement derived from the addition of an extra latent class. Proposed latent classes were examined for possible clinical interpretation and were assigned names based upon their presentation. This process is summarised in Additional file 2: Table S1.

Heterogeneity between flares and latent classes was assessed by ANOVA, and by t-tests with Bonferonni correction. Heterogeneity was sought for characteristics before, during or after the flare. Statistical analyses were performed using SPSS version 23 (IBM, USA), or, for LCA, using Mplus (Muthen and Muthen, Los Angeles, USA). Data from complete cases were used throughout, and no data imputation was performed.

## Results

DAS28 Flares were identified in 45% and Pain Flares in 52% of participants (Table 1). For participants with concurrent data permitting classification both of DAS28 Flare and Pain Flare, 60% (145/240) of participants at the time of their first DAS28 Flare did not concurrently fulfil criteria for a Pain Flare, and 64% (181/284) of participants at the time of their first Pain Flare did not concurrently fulfil criteria for a DAS28 Flare. The seropositive status of participants were similar in the overall eligible population and those recorded as having DAS28 Flares and Pain Flares (Table 1).

**Table 1:** Demographics of the eligible population at cohort baseline

	Categories or possible range	Eligible population	DAS28 Flare	Pain Flare
n		719	323	377
Sex	% Female	69%	68%	70%
Age (y)		56 (14)	57 (13)	56 (13)
Smoking	% Current Smoker	33%	30%	34%
	% Ex-smoker	24%	25%	26%
Body mass index (kg/m^2^)		27.7 (5.4)	27.5 (5.3)	27.8 (5.6)
DAS28-ESR		4.8 (1.5)	4.5 (1.6)	4.6 (1.6)
Erythrocyte Sedimentation Rate (mm/h)		30 (24)	28 (23)	31 (24)
Swollen joint count	0–28	6 (6)	6 (6)	6 (6)
Tender joint count	0–28	8 (7)	7 (7)	7 (7)
Visual analogue scale (mm)	0–100	46 (25)	43 (25)	44 (26)
Seropositive		58%	59%	58%
SF36 Bodily Pain	0–100	33 (11)	33 (11)	34 (11)
SF36 Vitality	0–100	41 (11)	42 (11)	42 (11)
SF36 Mental Health	0–100	46 (11)	47 (11)	47 (11)
HAQ	0–3	1.2 (0.9)	1.1 (0.8)	1.1 (0.7)

Baseline demographics at recruitment of participants who were eligible for this analysis by having at least 2 consecutive measurements of DAS28 and SF36-Bodily Pain (Eligible population) and the subgroups who were classified as experiencing flares. Seropositive; for rheumatoid factor or anti-citrullinated peptide according to local laboratory reference ranges. Mean (sd) or percent

**Fig. 1:**
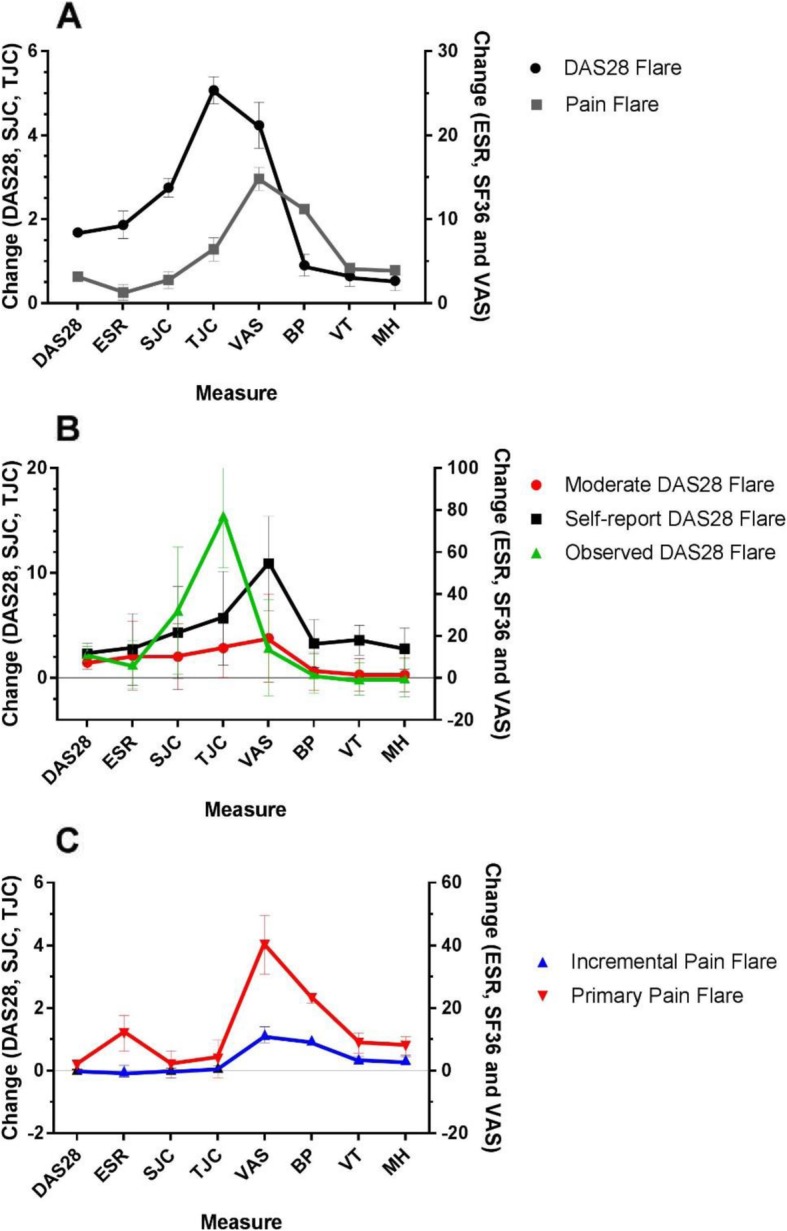
Characteristics of DAS28 Flares and Pain Flares. Data represent mean (95% CI) changes in each outcome at the time of flare compared to preceding assessment (note different scales in each panel). **a** DAS28 and Pain Flares; **b** subgroups of DAS28 Flare; **c** subgroups of Pain Flare. Y1 axis shows worsening change for BP=SF36-Bodily Pain, VT = SF36-Vitality, MH=SF36-Mental Health and VAS-GH. Y2 axis shows worsening change for SJC, TJC and DAS28

Demographics and clinical characteristics at the time of recruitment into the ERAN cohort did not differ to any clinically important extent between those who displayed either DAS28 or Pain Flare and the total eligible population (Table 1). The time to event analysis for first DAS28 Flare and first Pain Flare is shown on a Kaplan-Meier plot (Additional file 1: Figure S1). The first Pain flare occurred sooner than the first DAS28 flare (median (IQR), 4 (3–5) vs 6 (5–7) years; χ^2^ = 12.5, *p* = 0.0004). As expected, DAS28 Flares were associated with greater increases in DAS28, and in each DAS28 component, than were Pain Flares; whereas Pain Flares were associated with greater deteriorations in SF36 Bodily Pain score (Fig. 1a). Deteriorations in Vitality and Mental Health scores were similar during DAS28 or Pain Flares.

LCA identified 3 DAS28 Flare subgroups (Fig. 1b, Table 2). One DAS28 Flare subgroup (which we named ‘Observed DAS28 Flares’, 14% (34/236)) was characterised by greater increases in tender and swollen joint counts and a second subgroup (‘Self-report DAS28 Flares’, 13% (31/236)) was characterised by greater increases in patient-reported measurements including VAS-GH and tender joint count (Table 3). One DAS28 Flare subgroup (named ‘Moderate DAS28 Flares’, 72% (171/236)) displayed less severe deteriorations in outcome measures than did the other 2 subgroups. 36% of people with Moderate, and 27% with Observed DAS28 Flares concurrently fulfilled the Pain Flare criterion, whereas 79% of people with Self-report DAS28 Flares concurrently fulfilled the Pain Flare criterion. Observed DAS28 Flares tended to occur on a background of high pre-flare DAS28, symptoms and disability (Table 2).

**Table. 2:** Characteristics indicated by severity of change during DAS28 Flares

	All DAS28 Flares (*n* = 323)	Moderate DAS28 Flare (*n* = 171)	Self-report DAS28 Flare (*n* = 31)	Observed DAS28 Flare (*n* = 34)	*p*	Pairwise comparison between DAS28 Flare subgroups (*p* value)		
						M vs SR	M vs O	SR vs O
DAS28	1.7 (0.8)	1.5 (0.7)	**2.3 (1.0)**	**2.3 (0.7)**	< 0.001	< 0.001	< 0.001	0.999
ESR	9 (15)	11 (16)	13 (17)	7 (12)	0.206	0.999	0.510	0.247
SJC	3 (4)	2 (3)	**4 (4)**	**6 (6)**	< 0.001	0.011	< 0.001	0.100
TJC	5 (6)	3 (3)	6 (5)	**15 (5)**	< 0.001	< 0.001	< 0.001	< 0.001
VAS	21 (25)	19 (21)	**55 (23)**	14 (23)	< 0.001	< 0.001	0.801	< 0.001
BP	5 (10)	3 (9)	**16 (12)**	2 (9)	< 0.001	< 0.001	0.999	< 0.001
VT	3 (9)	2 (8)	**18 (7)**	−1 (8)	< 0.001	< 0.001	0.478	< 0.001
MH	3 (9)	1 (8)	**14 (10)**	0 (9)	< 0.001	< 0.001	0.999	< 0.001

Data are mean (sd) changes at the time of DAS28 Flare categorisation from the preceding assessment. Change during Flare is rescaled to indicate worsening with positive values for all measures (including SF36 subscales). Significant heterogeneity between latent classes was recorded after univariate ANOVA. Pairwise comparisons with p values (shown after Bonferonni corrections) were performed. MvSR = Moderate vs Self-report. MvO = Moderate vs Observed. SRvO=Self-report vs Observed. Bold indicates the values for the most severely affected subgroups when heterogeneity was detected

*ESR* erythrocyte sedimentation rate, *SJC* swollen joint count, *TJC* tender joint count, *VAS* visual analogue scale for general health, *BP* SF36-Bodily Pain, *VT* SF36-Vitality, *MH* SF36-Mental Health. Changes in SF36 scores = 10 indicate one standard deviation predicted for the UK population

**Table. 3:** DAS28 Flare subgroups and their characteristics at the visit before that at which they satisfied flare criteria

	Moderate DAS28 Flare (*n* = 171)	Self-report DAS28 flare (*n* = 31)	Observed DAS28 flare (*n* = 34)	*p*	Pairwise comparisons		
					MvSR	MvO	SRvO
DAS28	2.6 (1.1)	3.2 (1.3)	**4.0 (1.1)**	< 0.001	0.013	< 0.001	0.017
ESR	16 (13)	**26 (20)**	**22 (17)**	0.001	0.003	0.086	0.965
SJC	1 (2)	1 (3)	**3 (4)**	0.006	0.999	0.004	0.142
TJC	2 (4)	3 (4)	**5 (4)**	< 0.001	0.348	< 0.001	0.196
VAS	24 (18)	20 (16)	**45 (28)**	< 0.001	0.949	< 0.001	< 0.001
BP	40 (11)	42 (12)	**33 (11)**	0.001	0.999	0.002	0.005
VT	46 (11)	50 (9)	**36 (12)**	< 0.001	0.113	< 0.001	< 0.001
MH	50 (10)	52 (10)	**44 (11)**	0.005	0.999	0.009	0.012
HAQ	0.8 (0.8)	0.9 (0.8)	**1.4 (0.9)**	< 0.001	0.999	< 0.001	0.030

Data are mean (sd) of characteristics at the visit before flare criteria were satisfied. Significant heterogeneity between latent classes at the time point before flare categorisation was recorded after univariate ANOVA. Pairwise comparisons with *p* values (shown after Bonferonni corrections) were performed. *ESR* erythrocyte sedimentation rate, *SJC* swollen joint count, *TJC* tender joint count, *VAS* visual analogue scale for general health, *BP* SF36-Bodily Pain, *VT* SF36-Vitality, *MH* SF36-Mental Health. *SF36 scores* 50 indicate predicted population means, with lower scores indicating greater health impairment. *MvSR* Moderate vs Self-report, *MvO* Moderate vs Observed. *SRvO* Self-report vs Observed. Bold indicates the values for the most severely affected subgroups when heterogeneity was detected

**Fig. 2:**
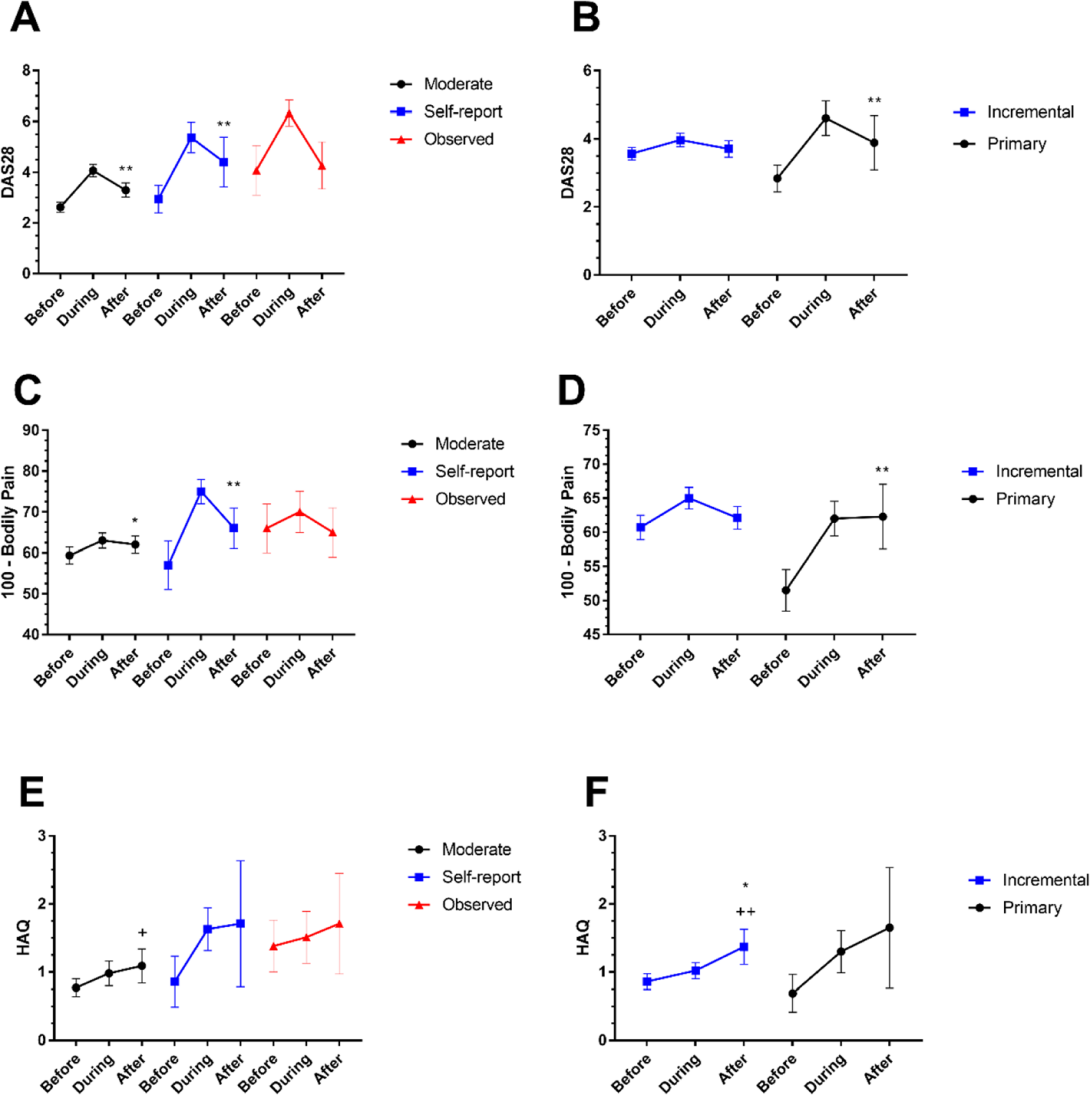
Changes in disease activity, pain and disability scores associated with DAS28 and Pain Flares. Mean (95% CI) of DAS28, SF36-Bodily Pain and HAQ scores at assessments before, during and after flare categorisation for DAS28 Flares (**a**, **c**, **e** respectively) and Pain Flares (**b**, **d**, **f** respectively). Statistical significant differences are denoted between during and after (** *p* < 0.01, * *p* < 0.05) and between before and after (++ *p* < 0.01 and + *p* < 0.05) flare categorisation

Two discrete Pain Flare subgroups were identified by LCA (Table 4, Fig. 1c) which differed in clinical severity both before (Table 5 and Fig. 2b, d, f) and during the flare (Table 4). One Pain Flare subgroup (which we named ‘Primary Pain Flares’, 11% (43/377)) displayed large increases in pain following a period of low disease activity and symptoms. The other Pain Flare subgroup (which we named ‘Incremental Pain Flares’, 89% (334/377)) occurred on the background of ongoing active disease and chronic pain (Table 5 and Fig. 2b, d, f). 77% of people with Primary Pain Flares but only 30% of people with Incremental Pain Flares concurrently fulfilled the DAS28 Flare criterion.

**Table 4:** Characteristics indicated by severity of change during Pain Flares

	All Pain Flares (*n* = 377)	Incremental Pain Flare (*n* = 334)	Primary Pain Flare (*n* = 43)	*p*
DAS28	0.6 (1.4)	0.3 (1.1)	**1.8 (1.4)**	< 0.001
ESR	1 (17)	0 (16)	**12 (18)**	< 0.001
SJC	1 (4)	0 (4)	**2 (4)**	0.012
TJC	1 (5)	1 (5)	**4 (6)**	0.001
VAS	15 (26)	11 (24)	**40 (30)**	< 0.001
BP	11 (6)	10 (4)	**23 (5)**	< 0.001
VT	4 (9)	4 (9)	**9 (10)**	0.001
MH	4 (10)	3 (10)	**8 (9)**	0.005

Data are mean (sd) changes at the time of Pain Flare categorisation from the preceding assessment. Change during flare is rescaled to indicate worsening with positive values for all measures (including SF36 subscales). Pairwise comparisons between latent classes of Pain Flare analysed by univariate t-tests. Bold indicates the values for the most severely affected subgroups when heterogeneity was detected

*ESR* erythrocyte sedimentation rate, *SJC* swollen joint count, *TJC* tender joint count, *VAS* visual analogue scale for general health, *BP* SF36-Bodily Pain, *VT* SF36-Vitality, *MH* SF36-Mental Health. Changes in SF36 scores = 10 indicate one standard deviation predicted for the UK population

**Table 5:** Pain Flare subgroups and their characteristics at the visit before that at which they satisfied flare criteria

	Incremental	Primary	*p*
	Pain Flare (*n* = 334)	Pain Flare (*n* = 43)	
DAS28	**3.6 (1.5)**	2.8 (1.3)	0.001
ESR	23 (19)	20 (16)	0.583
SJC	**3 (4)**	1 (3)	< 0.001
TJC	**5 (6)**	2 (3)	< 0.001
VAS	**30 (23)**	20 (19)	0.002
BP	**42 (10)**	50 (7)	< 0.001
VT	46 (11)	49 (13)	0.107
MH	50 (11)	53 (8)	0.134

Data are mean (sd) changes at the time of Pain Flare categorisation from the preceding assessment. Univariate t-tests for comparing the 2 classes of Pain flare at the time point before the flare. SF36 scores = 50 indicate predicted population means, with lower scores indicating greater health impairment. Bold indicates the values for the most severely affected subgroups when heterogeneity was detected

*ESR* erythrocyte sedimentation rate, *SJC* swollen joint count, *TJC* tender joint count, *VAS* visual analogue scale for general health, *BP* SF36-Bodily Pain, *VT* SF36-Vitality, *MH* SF36-Mental Health

We assessed whether flares were followed by continued worsening of RA severity (Fig. 2). Both DAS28 and Bodily Pain scores improved at the next assessment after the flare event, but not necessarily to the level which preceded the flare (Fig. 2). Most variables remained higher at the subsequent assessment after Self-report DAS28 Flare classification, but returned to pre-flare levels after Observed DAS28 flares. Average Bodily Pain scores did not return to pre-flare levels by the next assessment in either Pain Flare subgroup, and DAS28 did not return to pre-flare levels at the assessment following Primary Pain Flare classification. HAQ scores remained increased at the assessment following DAS28 or Pain Flares of any classification (Fig. 2). There were no significant differences observed in seropositive status of the flare subgroups for DAS28 Flare or Pain Flares.

## Discussion

We show flares in RA are common and heterogeneous. Flares of inflammatory disease activity and of pain often did not coincide. DAS28 and Pain Flares each comprised multiple discrete subgroups, characterised by increases in self-report symptoms or in observed synovitis, and occurring on different background disease activities or pain severity. DAS28 and Pain typically improved after flares, but disability was persistently increased. Flares represent a substantial burden for people with RA, and might contribute to progressive functional decline. Distinguishing different flare types should inform personalised treatment.

RA is a chronic inflammatory disease subject to relapse and remission. We show that RA flares are common and heterogeneous. Patient characteristics were similar between those experiencing DAS28 or Pain Flares, each occurred on a background of similar levels of acute phase response, and each was accompanied by similar deteriorations in fatigue, mental health and disability. Different flare types might therefore have similar impact and affect any individual with RA. However, DAS28 and Pain Flare episodes differ in severity of observed synovitis or patient-reported pain during the flare episode, and often did not coincide. Our findings indicate that DAS28-defined flares alone might not represent the breadth of flare experience in RA and an alternative, patient-centred classification of RA Pain Flare identifies episodes that differ from DAS28 Flares. We show that DAS28 and Pain Flares can be further subdivided into discrete classes, further emphasising the heterogeneous nature of the RA experience.

Different flare types might represent different aspects of RA pathophysiology. Inflammation causes joint pain, and RA flares have been defined in terms of increases in DAS28 [3]. However, RA pain may be due to mechanisms other than inflammation, including central sensitisation or joint damage [19]. Discordance between pain and signs of inflammation have been noted in previous cross sectional studies of RA [8, 20, 21]. Our findings indicate that discordance between inflammation and pain is also characteristic of flare events. Self-report DAS28 Flares resemble the previously described ‘discordant’ group of people with active RA, who display high self-report but relatively low observed disease activity measures [8]. Such discordance has been associated with central sensitisation or fibromyalgia [20, 22].

DAS28 and Pain typically improved after flares, but disability was persistently increased after any type of flare studied. In the self-report DAS28 Flare subgroup, DAS28 and Pain also remained persistently increased after flare, and pain also remained persistently increased after Pain Flare classification. Flares across different subtypes therefore can have long term detrimental effects. Flares can promote also disease progression [6, 23, 24], and inflammation can lead to central sensitisation [25] which contributes to persistent pain. Changes in activity, domestic responsibilities, or employment during flares might lead to longer term behavioural and functional changes that contribute to persistent disability.

Patients often present to health care providers during flares, and treatment aims to both relieve current symptoms and prevent long term sequelae. Different strategies might be targeted to prevent or treat different flare types. Flare treatment often focuses on suppressing inflammatory disease, for example by glucocorticoid administration or DMARD escalation, and this strategy might be most effective where there is objective evidence of synovitis. RA inflammatory disease may be active and continue to damage joints even in the absence of notable pain [26]. Non-inflammatory RA pain is associated with less joint damage [27] and might still be resistant to treatment by immunosupression or anti-inflammatory drugs [28, 29]. Self-report DAS28 and Primary Pain Flares were also associated with worse mental health scores, suggesting that treatment of psychological comorbidity might be indicated. Effective disease suppression might reduce flares, but suppression of inflammation alone might not be sufficient to prevent disability progression. Adjunctive medical, physiotherapeutic or occupational approaches might be necessary to improve outcomes in people who experience flares.

Our study is subject to a number of important limitations. Flares will have occurred and resolved between assessments in the ERAN cohort, although the per protocol assessment schedule in ERAN might mean that flares classified during ERAN assessments were representative of RA flares. Assessments might not have captured data at a flare’s peak, and pre-flare data might not reflect the patient’s best clinical status. Our data therefore are likely to underestimate the prevalence of flares in this RA population. Our definitions of flare have evidence of validity [2], but we do not know whether patients or physicians considered participants to be in flare at the time of our flare classification. Our criterion for Pain Flares was validated against OMERACT flare classification and concurs with Minimal Clinically Important Differences in pain [30]. Pain Flares were not validated directly against patient self-reported flare, but displayed similar overall severity to DAS28 Flares. Identification of flare subgroups depends upon the variables entered into latent class models, and it is possible that additional heterogeneity exists within our identified flare subgroups. For example, fatigue behaved similarly between our flare subgroups, although flares of fatigue might also display discrete subgroups [7]. It is also possible that non-random patterns of missing data might have influenced the study findings. There were more missing data in analyses of DAS28 Flares than in Pain Flares. One of the contributing reasons was that some people had data for DAS28 recorded but without the 4 components. DAS28 Flare classification incorporates components measuring both symptoms and signs of inflammation [31, 32]. More objective measures of synovitis such as ultrasound imaging might reveal greater separation between inflammatory and pain flare subtypes. It is also possible the omission of the VAS from the DAS28 might also show more separation of inflammatory and pain flare subtypes. Measurement error might have contributed to some flare classifications, although the magnitudes of change that were recorded are regarded as clinically important. We have also not been able to analyse how DMARD medication choices might control or contribute to flares, and future randomised controlled trials might usefully explore flare reduction as a clinically relevant outcome of treatment.

## Conclusion

In conclusion, we show that flares in RA are common and heterogeneous. DAS28 and Pain Flares are discrete entities indicative of differing underlying mechanisms, but both have immediate patient impact and lead to longer term disability. Identifying and understanding RA flare subgroups should guide treatment. Due to their intermittent nature, flares might go unnoticed between regular reviews, but represent a substantial burden for people with RA, and might contribute to progressive functional decline. Treatments are needed to reduce flare incidence as well as supressing flares when they happen, and should aim to reduce long term disability and pain, as well as improving current symptoms**.**

## Supplementary information

**Additional file 1: Figure S1.** Time until first DAS28 Flare and first Pain Flare. Kaplan-Meier plot of time until the first flare.

**Additional file 2: Table S1.** Selection of latent classes of flare. Summary of the indices used to select subtypes of DAS28 flare and Pain flare.

## Data Availability

The datasets generated and/or analysed during this study are not publically available, but can be obtained via a reasonable request to the ERAN Steering Committee, via the Corresponding author.

## Abbreviations

ACR: American College of Rheumatology.
AIC: Akaike Information Criteria
BIC: Bayesian Information Criteria
BLRT: Bootstrap likelihood ratio test
BMI: Body mass index
CI: Confidence interval
DAS28: 28 joint disease activity score
DMARD: Disease-modifying anti-rheumatic drug
ERAN: Early Rheumatoid Arthritis Network
ESR: Erythrocyte sedimentation rate
HAQ: Health assessment questionnaire
LCA: Latent Class Analysis
LMR-LRT: Lo-Mendell-Rubin likelihood ratio test
RA: Rheumatoid arthritis
RF: Rheumatoid factor
SD: Standard deviation
SF36: Short Form-36 questionnaire
SJC: Swollen joint count
SSBIC: Sample-size adjusted BIC
TJC: Tender joint count
VAS: Visual analogue scale
VAS-GH: Visual analogue scale-general health
