# Supplementary material for: Disease activity flares and pain flares in an early rheumatoid arthritis inception cohort; characteristics, antecedents and sequelae
Source: BMC Rheumatol. 2019 Nov 18;3:49. doi: 10.1186/s41927-019-0100-9 (PMC6859633; doi:10.1186/s41927-019-0100-9)
Supplement: Supplementary file 1 — Additional file 1: Figure S1. Time until first DAS28 Flare and first Pain Flare. Kaplan-Meier plot of time until the first flare. [file 41927_2019_100_MOESM1_ESM.docx]

Supplement Figure 1: Time until first DAS28 Flare and first Pain Flare

Kaplan-Meir plot of time until first flare event for DAS28 Flares and Pain Flares.
