# Supplementary material for: Disease activity flares and pain flares in an early rheumatoid arthritis inception cohort; characteristics, antecedents and sequelae
Source: BMC Rheumatol. 2019 Nov 18;3:49. doi: 10.1186/s41927-019-0100-9 (PMC6859633; doi:10.1186/s41927-019-0100-9)
Supplement: Supplementary file 2 — Additional file 2: Table S1. Selection of latent classes of flare. Summary of the indices used to select subtypes of DAS28 flare and Pain flare. [file 41927_2019_100_MOESM2_ESM.docx]

Supplement Table 1: Selection of latent classes of flare

|  | AIC | BIC | ssBIC | Entropy | Largest % | Second % | Third % | Fourth % | VLMR (p) | BLRT (p) |
| --- | --- | --- | --- | --- | --- | --- | --- | --- | --- | --- |
| DAS28 flare classes | | | |  |  |  |  |  |  |  |
| 1 | 12259 | 12307 | 12263 |  | 100% |  |  |  |  |  |
| 2 | 12157 | 12234 | 12164 | 0.804 | 85% | 15% |  |  | 114.8 (0.0652) | 117.4 (<0.0001) |
| **3** | **12081** | **12185** | **12090** | **0.863** | **74%** | **14%** | **12%** |  | **90.7 (0.0530)** | **92.8 (<0.0001)** |
| 4 | 12035 | 12167 | 12046 | 0.89 | 73% | 14% | 11% | 2% | 60.2 (0.0626) | 61.6 (<0.0001) |
|  |  |  |  |  |  |  |  |  |  |  |
| Pain flare classes | | | |  |  |  |  |  |  |  |
| 1 | 15695 | 15747 | 15703 |  | 100% |  |  |  |  |  |
| **2** | **15546** | **15628** | **15558** | **0.918** | **88%** | **12%** |  |  | **161.8 (0.0146)** | **165.3 (<0.0001)** |
| 3 | 15473 | 15585 | 15490 | 0.937 | 84% | 11% | 4% |  | 86.3 (0.1048) | 88.2 (<0.0001) |
| 4 | 15427 | 15568 | 15448 | 0.942 | 81% | 11% | 5% | 3% | 61.3 (0.3400) | 62.7 (<0.0001) |

Summary of the indices used to select subtypes of DAS28 flare and Pain flare. AIC = Akaike Information Criteria, BIC = Bayesian Information Criteria, ssBIC=sample size adjusted Bayesian Information Criteria, VLMR=Vuo Lu Mendell Rubin likelihood ratio test, BLRT = Bootstrapping Likelihood Ratio Test. The proportion of the sample that is taken by each latent class is displayed in order of size.
